# Combined administration of testosterone plus an ornithine decarboxylase inhibitor as a selective prostate-sparing anabolic therapy

**DOI:** 10.1111/acel.12174

**Published:** 2013-12-04

**Authors:** Ravi Jasuja, James C Costello, Rajan Singh, Vandana Gupta, Catherine S Spina, Gianluca Toraldo, Hyeran Jang, Hu Li, Carlo Serra, Wen Guo, Pratibha Chauhan, Navjot S Narula, Tyler Guarneri, Ayla Ergun, Thomas G Travison, James J Collins, Shalender Bhasin

**Affiliations:** 1 Research Program in Men’s Health: Aging and Metabolism, Boston Claude D. Pepper Older Americans Independence Center for Function, Promoting Anabolic Therapies, Brigham and Women’s Hospital, Harvard Medical School221 Longwood Avenue, Boston, MA, 02115, USA; 2 Howards Hughes Medical Institute, Center for BioDynamics, Boston UniversityBoston, MA, 02115, USA; 3 Division of Endocrinology and Metabolism, Charles Drew University of Medicine and Science, David Geffen School of Medicine at UCLALos Angeles, CA, 90059, USA; 4 Wyss Institute for Biologically Inspired Engineering, Harvard UniversityBoston, MA, 02215, USA

**Keywords:** aging, anti-aging, sarcopenia, sex hormones, skeletal muscle, steroids

## Abstract

Because of its anabolic effects on muscle, testosterone is being explored as a function-promoting anabolic therapy for functional limitations associated with aging; however, concerns about testosterone’s adverse effects on prostate have inspired efforts to develop strategies that selectively increase muscle mass while sparing the prostate. Testosterone’s promyogenic effects are mediated through upregulation of follistatin. We show here that the administration of recombinant follistatin (rFst) increased muscle mass in mice, but had no effect on prostate mass. Consistent with the results of rFst administration, follistatin transgenic mice with constitutively elevated follistatin levels displayed greater muscle mass than controls, but had similar prostate weights. To elucidate signaling pathways regulated differentially by testosterone and rFst in prostate and muscle, we performed microarray analysis of mRNAs from prostate and *levator ani* of castrated male mice treated with vehicle, testosterone, or rFst. Testosterone and rFst shared the regulation of many transcripts in *levator ani;* however, in prostate, 593 transcripts in several growth-promoting pathways were differentially expressed after testosterone treatment, while rFst showed a negligible effect with only 9 transcripts differentially expressed. Among pathways that were differentially responsive to testosterone in prostate, we identified ornithine decarboxylase (Odc1), an enzyme in polyamine biosynthesis, as a testosterone-responsive gene that is unresponsive to rFst. Accordingly, we administered testosterone with and without α-difluoromethylornithine (DFMO), an Odc1 inhibitor, to castrated mice. DFMO selectively blocked testosterone’s effects on prostate, but did not affect testosterone’s anabolic effects on muscle. Co-administration of testosterone and Odc1 inhibitor presents a novel therapeutic strategy for prostate-sparing anabolic therapy.

## Introduction

Testosterone’s anabolic effects on the skeletal muscle are widely recognized (Bhasin *et al*., 2006). Testosterone levels in men are positively associated with skeletal muscle mass, strength, and physical function (Roy *et al*., 2002; Orwoll *et al*., 2006). Consistent with these epidemiological correlations, testosterone administration also increases lean body mass and maximal voluntary strength in men (Bhasin *et al*., 1996; Bhasin *et al*., 1997; Snyder *et al*., 1999; Wang *et al*., 2000; Page *et al*., 2005; Srinivas-Shankar *et al*., 2010). A number of androgens, including testosterone, are being investigated as function-promoting anabolic therapies for the treatment of functional limitations associated with aging and chronic illness (Bhasin *et al*., 2006). However, concerns about the potential prostatic side effects of testosterone have limited enthusiasm for testosterone’s applications as a function-promoting anabolic therapy in older men (Bhasin *et al*., 2006; Calof *et al*., 2005). Thus, there is an unmet need for novel therapeutics that selectively exert anabolic effects on the muscle, but which are free of adverse effects on prostate (Bhasin *et al*., 2006; Dalton *et al*., 2011; Basaria *et al*., 2013). Here, we report a novel strategy for achieving the selectivity of testosterone’s anabolic actions on skeletal muscle while sparing the prostate.

We have reported that testosterone promotes myogenic differentiation of muscle progenitor cells by activating AR/β-catenin pathway and inducing the expression of a number of Wnt-target genes, including follistatin (Singh *et al*., 2009; Braga *et al*., 2012). Follistatin is essential for mediating testosterone’s effects on myogenesis; blocking follistatin’s action using either antifollistatin antibodies or siRNAs blocks testosterone’s effects on myogenic differentiation of muscle progenitor cells (Singh *et al*., 2009; Braga *et al*., 2012). These data suggested that testosterone and follistatin likely activate analogous pathways in the muscle. We show here that the administration of recombinant follistatin (rFst) increased muscle mass, as expected (Lee, 2007; Kota *et al*., 2009; Singh *et al*., 2009; Dalton *et al*., 2011; Braga *et al*., 2012), but surprisingly, unlike testosterone, rFst did not affect prostate mass. These data suggested that in prostate, testosterone activates signaling pathways that contribute to prostate growth, but which are not activated by follistatin.

To identify signaling pathways in the prostate that are activated selectively by testosterone, we performed microarray analysis of mRNAs in prostate and *levator ani* of castrated mice treated with placebo, testosterone, or rFst. Among biological processes that were differentially activated in prostate by testosterone, but not by follistatin, we identified polyamine biosynthesis as a key androgen-sensitive pathway that is known to regulate prostate growth (Pegg & Williams-Ashman, 1968; Danzin *et al*., 1982). Ornithine decarboxylase (Odc1), the rate-limiting enzyme in polyamine biosynthesis, was differentially expressed in the prostate glands of testosterone-treated mice, while showing no significant change in rFst-treated mice. We reasoned that if Odc1 is essential for mediating testosterone’s effects on the prostate, but not the muscle, then the administration of testosterone plus Odc1 inhibitor should block testosterone’s effects on prostate mass, but not on muscle mass. Indeed, we show here that combined administration of testosterone plus α-difluoromethylornithine (DFMO), an irreversible Odc1 inhibitor, increased muscle mass, but did not induce prostatic growth. Thus, combination of testosterone plus an Odc1 inhibitor provides a novel therapeutic approach for achieving the selectivity of testosterone’s effects on the muscle while sparing the prostate.

## Results

### rFst increases lean mass and decreases fat mass without affecting prostate growth

Previous studies have shown that the effects of testosterone on differentiation of mesenchymal progenitor cells are mediated through follistatin (Singh *et al*., 2009; Braga *et al*., 2012). These observations suggested that follistatin might exert effects on androgen-responsive tissues, such as the muscle, fat, and prostate, similar to those of testosterone. Indeed, follistatin has been shown to increase skeletal muscle mass (Lee, 2007; Kota *et al*., 2009); however, the data on its effects on the prostate and adipose tissue have been discordant (McPherson *et al*., 1997; Hirai *et al*., 2007; Flanagan *et al*., 2009; Tumminello *et al*., 2010; Nakatani *et al*., 2011). Higher circulating follistatin levels have been correlated with increased fat mass (Flanagan *et al*., 2009); follistatin has also been reported to induce adipogenic differentiation of preadipocytes (Hirai *et al*., 2007). However, transgenic expression of a follistatin peptide in mice was associated with reduced fat accumulation (Tumminello *et al*., 2010). Follistatin neutralizes activin-mediated suppression of prostate cell growth (McPherson *et al*., 1997); furthermore, follistatin levels in men with prostate cancer have been associated with increased risk of bone metastasis (Nakatani *et al*., 2011).

**Figure 1:**
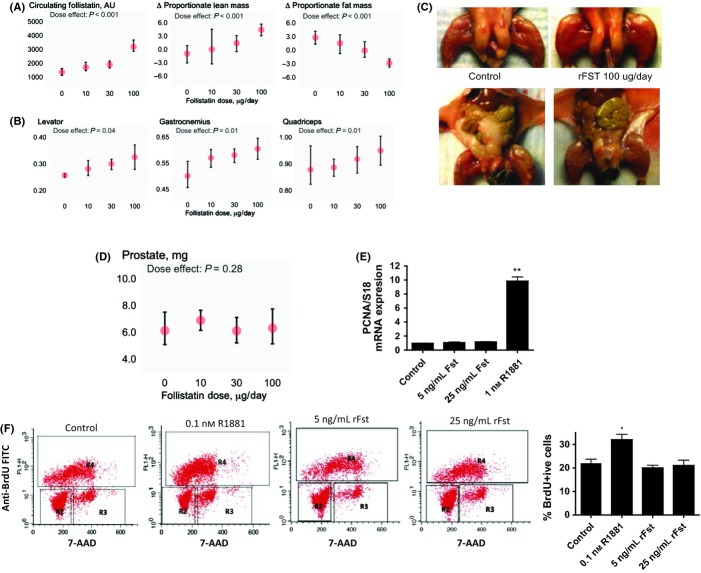
Recombinant follistatin (rFst) increases lean mass and decreases fat mass without affecting prostate growth. (A) rFst supplementation dose dependently increases circulating follistatin levels. Changes in percent lean body mass (middle panel) and fat mass (right panel) were related to rFst dose (*P* < 0.0001). Data are mean ± SEM, *n* = 6 in each group. (B) Mean ± SEM wet weights of *levator ani*, *gastrocnemius*, and *quadriceps femoris* were significantly higher in mice treated with 100 μg rFst daily than in vehicle-treated mice. *n* = 6 in each group. (C) Marked hypertrophy of lower extremity muscles and a reduction in intra-abdominal fat mass in 8-week-old male mice treated with vehicle (rFst 0) or 100 μg of rFst (rFst 100) daily for 13 weeks. (D) Mean ± SEM prostate weights in mice treated with 0, 10, 30, and 100 μg rFst daily for 13 weeks. Prostate weights did not differ between animals treated with rFst or vehicle. (E) rFst does not affect PCNA expression in primary prostate epithelial cells. Primary prostate epithelial cells, synchronized for 24 h, were incubated with 0, 5, or 25 ng mL^−1^ rFst or 1 nm R1881, an androgen. PCNA mRNA expression, measured using quantitative PCR and normalized against 18S ribosomes, was upregulated nearly 10-fold by R1881, but was unaffected by rFst even at concentrations that were nearly 100 times the circulating follistatin levels in humans. (F) rFst supplementation does not affect BrdU incorporation or the fraction of LnCaP cells in the S phase. The panels show the flow cytometric profile; R2, R3, and R4 represent the fraction of cells in G1, S, and G2/M phases, respectively. While R1881 even at concentrations as low as 0.1 nm increased the percent of cells in the S phase and the percent of BrdU+ cells, neither 5 nor 25 ng mL^−1^ rFst had any significant effect on the percent of BrdU+ cells. *P < 0.05; **P < 0.01 vs. control.

To determine the effects of follistatin on androgen-responsive tissues – muscle, fat, and the prostate, we expressed rFst protein and administered graded doses of rFst to C57BL6 adult male mice (Fig. 1). rFst administration was associated with dose-dependent increases in circulating follistatin levels and lean body mass, measured using nuclear magnetic resonance (Fig. 1A). The wet weights of *levator ani*, *gastrocnemius*, and *quadriceps femoris* muscle groups were related to rFst dose (Fig. 1B) and were significantly higher in mice treated with 100 μg rFst daily than in vehicle-treated mice. rFst administration was associated with a dose-dependent reduction in whole-body and intra-abdominal fat mass (Fig. 1A,C).

Surprisingly, rFst administration did not significantly affect prostate weight (Fig. 1D). Even in mice receiving the highest dose of rFst (100 μg daily), the mean prostate weight was not significantly different from that in vehicle-treated mice, while the *levator ani* weight was 25% higher than in vehicle-treated controls.

To further characterize the effect of rFst on the growth of prostate cells, we incubated androgen-responsive primary prostate epithelial cells with 0, 5, or 25 ng mL^−1^ rFst or with methyltrienolone (R1881), a synthetic nonaromatizable androgen (Fig. 1E). As expected, R1881 upregulated the mRNA levels of cell growth marker PCNA, but rFst had no effect on PCNA expression even at concentrations that were nearly 100-fold higher than those in human circulation (O’Connor *et al*., 1998).

Additionally, we examined the effects of rFST and an androgen, R1881, on the proportion of androgen-sensitive LNCaP cells in S phase in cell cycle analysis using BrdU incorporation combined with DNA intercalation dye 7-AAD. The cell cycle phases of actively dividing LNCaP cells (BrdU+) were resolved using fluorescence-activated cell sorting. Unlike R1881, which increased BrdU incorporation as well as the fraction of LNCaP cells in S phase at concentrations as low as 0.1 nm, rFst had no significant effect on either the percent of BrdU+ cells or the fraction of cells in S phase (Fig. 1F).

### Differential effects of follistatin hyperexpression on skeletal muscle mass and prostate in follistatin transgenic mice

**Figure 2:**
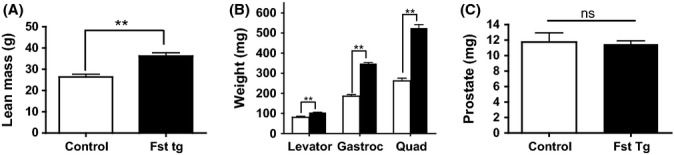
Differential effects of follistatin hyperexpression on skeletal muscle mass and prostate in follistatin transgenic mice. (A) Follistatin transgenic mice (Fst Tg) had significantly higher lean body mass than wild-type controls. (B) Follistatin transgenic mice had higher weights of *levator ani*, *gastrocnemius,* and *quadriceps femoris* than wild-type controls. (C) Prostate weights did not differ significantly between follistatin transgenic mice and wild-type controls. **P < 0.05 vs. wild-type controls.

Follistatin transgenic mice, in which higher circulating levels of follistatin are derived from its constitutive overexpression in skeletal muscle (Lee, 2007), had higher lean mass than their wild-type littermates (Fig. 2A). The wet weights of *levator ani*, *gastrocnemius*, and *quadriceps* were also significantly higher in follistatin transgenic mice than in wild-type controls (Fig. 2B), even after adjusting for body weights. However, prostate weights did not differ significantly between the follistatin transgenic and wild-type mice (Fig. 2C). These data further support the notion that follistatin selectively promotes muscle growth, but spares the prostate.

### Microarray analysis of genes and pathways differentially regulated by testosterone and rFst in the muscle and prostate

As follistatin is in the signaling pathway that mediates the effects of testosterone on myogenesis, the observations that rFst selectively increased skeletal muscle mass, but did not affect prostate growth or the markers of prostate cell proliferation *in vitro*, suggested that testosterone differentially activates specific signaling pathways in the prostate that are not activated by rFst. We surmised that signaling pathways that are activated in prostate by testosterone, but not by rFst, are the likely mediators of testosterone’s effects on the prostate and would be of interest with respect to developing therapeutic strategies for achieving the selectivity of testosterone’s actions.

Accordingly, we used microarray analysis to characterize the transcriptome of *levator ani* and prostate under the following conditions: (i) intact male mice, (ii) castrated male mice treated with vehicle alone, (iii) castrated male mice treated with testosterone, and (iv) castrated male mice treated with rFst. A minimum of four replicates were run for each group. A gene was considered androgen sensitive if it was differentially expressed at a *q*-value < 0.2 comparing intact to castrated mice and castrated mice to testosterone-treated castrated mice. Similarly, a gene was considered follistatin sensitive if it was differentially expressed at a *q*-value < 0.2 comparing intact to castrated mice and castrated mice to follistatin-treated castrated mice. After compiling the lists of genes classified as androgen sensitive and follistatin sensitive, Gene Ontology (GO) term enrichment analysis was performed to identify GO terms over-represented in either category (Table S1).

**Figure 3:**
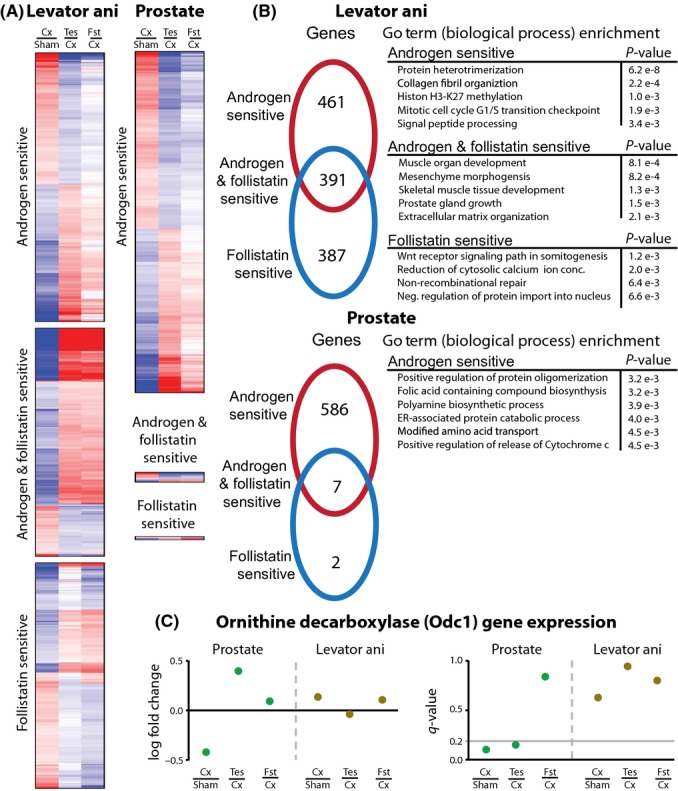
Microarray analysis of testosterone and rFst in the muscle and prostate. (A) The heatplots represent the statistically significant genes (*q*-value < 0.2) in the *levator ani* and prostate for the listed experimental condition comparisons across untreated (Sham), castrated (Cx), testosterone-treated (Tes), and follistatin-treated (Fst) mice. Heatplots were classified into the following groups: uniquely androgen sensitive, uniquely follistatin sensitive, and both androgen and follistatin sensitive. (B) The venn diagram represents the number of genes that are differentially expressed in the categories taken from the heatplots. Gene Ontology (GO) term enrichment analysis was performed on the associated sets of genes. Selected GO terms that are significant at an adjusted *P*-value < e^−3^ are listed. Follistatin treatment has a negligible effect on the prostate and no enriched GO terms. (C) Gene expression ratios (log_2_ fold change) and the statistical significance of the ratios (*q*-value) are shown for ornithine decarboxylase (Odc1). Odc1 is androgen sensitive, but not follistatin sensitive, in the prostate, and no effect is seen in the *levator ani*.

There were 852 genes classified as androgen sensitive and 778 classified as follistatin sensitive in the *levator ani* (Fig. 3A,B). Of these genes, 391 were found to be both androgen and follistatin sensitive. As expected from the anabolic effects of both testosterone and follistatin on skeletal muscle, GO term enrichment revealed a large number of biological processes related to cell growth and development (Fig. 3B).

Microarray analysis of prostate yielded results that were strikingly different from those in the muscle (Fig. 3A–C). We identified 593 androgen-sensitive genes, but only 9 follistatin-sensitive genes. Of the nine follistatin-sensitive genes, seven were also androgen sensitive. These microarray analyses are consistent with the observations that testosterone promotes the growth of both muscle and prostate, while rFst only affects muscle growth, but spares the prostate.

The microarray data provide a basis from which we can identify biological processes that can be targeted to reduce the androgenic effects of testosterone on the prostate. Among the biological pathways that were activated in the prostate selectively by testosterone (Fig. 3B; Fig. S1), we identified polyamine biosynthetic pathway as a key target for several reasons. First, polyamine synthesis was androgen sensitive, but not follistatin sensitive, in the prostate; this pathway was not among those differentially induced by testosterone and rFst in *levator ani*. Second, polyamine biosynthesis is known to be important in the regulation of prostate growth (Pegg & Williams-Ashman, 1968; Danzin *et al*., 1982). Third, ornithine decarboxylase (Odc1), a key enzyme in polyamine biosynthesis, exhibited an expression signature indicative of its differential regulation by testosterone in the prostate (Fig. 3C, Fig. S2). Selective upregulation of Odc1 by testosterone, but not by rFST, was confirmed in LNCaP cells treated with 100 nm testosterone or 125 ng mL^−1^ rFst over a period of 24 h (Fig. S3). A time-course evaluation of testosterone supplementation to castrated mice exhibited time-dependent upregulation of Odc1; there was a close concordance between prostate weight recovery and the increase in Odc1 expression in response to testosterone administration (Figs S4 and S5).

Odc1 was significantly downregulated upon castration in the prostate, and its expression was restored after testosterone administration, but not after rFst administration. In the *levator ani*, Odc1 expression showed little or no difference in its expression levels under any treatment (Fig. 3C). Finally, Odc1 catalyzes the decarboxylation of L-ornithine to form putrescine, which is known to be important in the regulation of prostate growth (Pegg & Williams-Ashman, 1968; Danzin *et al*., 1982; Pegg, 1988). Our data combined with the previous characterization of Odc1 as a molecule that is important in prostate growth and is androgen sensitive led us to hypothesize that Odc1 may be the basis of the differential sensitivity of the prostate to testosterone and rFst.

### Inhibition of Odc1 by DFMO blocks testosterone’s effects on the prostate while retaining its anabolic effects on the muscle

We reasoned that if Odc1 were essential for mediating testosterone’s effects on the prostate, but not the muscle, then the inhibition of Odc1 activity would be expected to attenuate the prostate-stimulating effects of testosterone while retaining its anabolic effects on the muscle. Accordingly, adult male intact and castrated mice were treated for 2 weeks with vehicle or testosterone with and without DFMO, a specific Odc1 inhibitor, as follows: intact (I), castrated (Cx), castrated + 15 μg per day testosterone (Cx + T), and castrated +15 μg per day testosterone + 15 μg per day DFMO (Cx + T + DFMO). The doses and duration of DFMO treatment were based on previous studies that demonstrated effective inhibition of ODC activity *in vivo* (Danzin *et al*., 1982).

**Figure 4:**
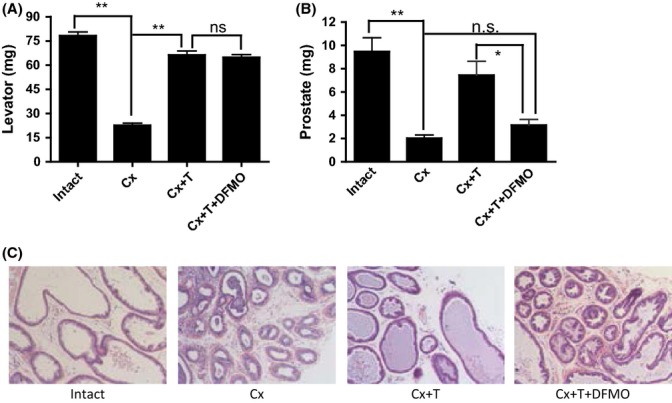
Inhibition of Odc1 by α-difluoromethylornithine (DFMO) blocks testosterone’s effects on the prostate while retaining its anabolic effects on the muscle. Adult male intact and castrated mice were treated for 2 weeks with vehicle or testosterone with and without DFMO, a specific Odc1 inhibitor, as follows: intact, castrated (Cx), castrated + 15 μg per day T (Cx + T), castrated + 15 μg per day T + 15 μg per day DFMO (Cx + T + DFMO). (A) *levator ani* weights in mice treated with testosterone plus DFMO were similar to those in intact controls and testosterone-treated castrated mice. (B) Prostate weights in castrated mice were lower than in intact controls and were restored by testosterone administration to levels seen in intact mice. Mice treated with testosterone plus DFMO had significantly lower prostate weights than intact controls or castrated mice treated with testosterone alone, but not significantly different from those in castrated mice treated with vehicle alone. (C) Histological sections of prostate reveal marked involution of prostate glands in castrated mice, but not in testosterone-treated castrated mice. Administration of testosterone plus DFMO in castrated mice failed to prevent castration-associated involution of prostate glands and changes in epithelial layers. Nine sections per group were examined, and representative images are shown *P < 0.05; **P < 0.01.

As expected, castrated mice had significantly lower *levator ani* weight as well as lower prostate weight than intact controls (Fig. 4A,B). Testosterone administration alone in castrated mice restored the weights of prostate and *levator ani* muscle to those observed in intact eugonadal controls. Importantly, combined administration of testosterone plus DFMO restored *levator ani* mass to that of intact controls and testosterone-treated castrated mice. However, prostate weights in castrated mice treated with testosterone plus DFMO were significantly lower than those of intact controls. Furthermore, testosterone administration prevented the involution of prostate gland and lumen observed in castrated mice; co-administration of DFMO blocked the testosterone’s effect on prostate epithelial growth (Fig. 4C). Thus, Odc1 is essential for mediating testosterone’s effects on the prostate, but not muscle.

## Discussion

The past decade has witnessed enormous pharmaceutical interest in the development of selective androgen receptor modulators (SARMs) that have preferential anabolic effects on the muscle, but which are relatively selective with respect to the prostate (Bhasin *et al*., 2006; Dalton *et al*., 2011; Basaria *et al*., 2013). We report here two novel therapeutic strategies for achieving the selectivity of anabolic effects on the skeletal muscle while sparing the prostate. We show that Odc1, a rate-limiting enzyme in the polyamine biosynthetic pathway, is an important mediator of testosterone’s effects on the prostate and that the administration of testosterone in combination with an Odc1 inhibitor blocks testosterone’s effects on the prostate, but not on the muscle. Additionally, we show here that follistatin, a mediator of testosterone’s effects on myogenesis, selectively increases skeletal muscle mass without affecting the prostate. Although both strategies could potentially exert anabolic effects while sparing the prostate, the testosterone plus Odc1 inhibitor combination could be attractive in patient populations in whom improvements in sexual function may be a desired outcome. Furthermore, testosterone and DFMO have been administered previously to humans and approved by FDA for other indications; therefore, they provide an accelerated path to clinical application.

Ornithine decarboxylase is a widely distributed enzyme that is expressed in the prostate as well as in the skeletal muscle (Pegg & Williams-Ashman, 1968; Pegg, 1988; Jänne *et al*., 1991; Lee *et al*., 2011). Polyamine synthesis has been implicated in the regulation of prostate growth (Pegg, 1988; Jänne *et al*., 1991). The enzyme, ornithine decarboxylase, the first enzyme in the polyamine synthesis that converts ornithine to putrescine, has been shown to interact directly with androgen receptor, contains an AR-binding site in its promoter, and plays an important role in the regulation of prostate growth (Pegg & Williams-Ashman, 1968; Danzin *et al*., 1982). The polyamines are essential for proliferating cells (Pegg, 1988), especially in the prostate. Testosterone has been reported to regulate ornithine decarboxylase in the muscle and prostate (Jänne *et al*., 1991). Our data demonstrate that Odc1 is essential for mediating testosterone’s effects on the prostate, but not on the muscle. These observations suggest that the combination of testosterone plus DFMO or other Odc1 inhibitors can serve as selective androgen therapy that spares the prostate. Odc1 is an attractive therapeutic target due to its short half-life and because a number of Odc1 inhibitors have been developed previously for other indications (Abeloff *et al*., 1984; Boitz *et al*., 2009). Separately, testosterone and DFMO each have been administered previously to humans for other indications. Testosterone alone has been used for the treatment of hypogonadism in men, while DFMO alone has been used for the treatment of leishmaniasis, hirsutism, and other disorders (Abeloff *et al*., 1984; Bhasin *et al*., 2006; Boitz *et al*., 2009). Preclinical and clinical data on their safety when they are administered alone are available. Thus, the combination of testosterone plus DFMO combination could proceed to clinical trials with more limited preclinical toxicology studies than are typically required for completely new molecules.

While we have focused on the Odc1 pathway because of its known role in prostate growth, it is possible that additional pathways may play an important direct or indirect role in mediating testosterone’s effects on the prostate. In addition to the prostate and muscle, testosterone regulates other physiological processes, including erythropoiesis, bone health, secondary sex characteristics, and sexual function. While we have shown that the Odc1 inhibitor, DFMO, blocks the prostate effects of testosterone without attenuating its anabolic effects on the muscle, additional studies are needed to ensure that other physiological effects of testosterone, especially on sexual function and bone, are not attenuated by the administration of an Odc1 inhibitor.

This drug combination would be particularly attractive for the treatment of conditions in which testosterone therapy alone may be associated with a high risk of adverse prostate events, such as older men with low testosterone levels, hypogonadal men at increased risk of prostate cancer, or men with prostate cancer who have undergone radical prostatectomy and are at low risk of disease recurrence. Accordingly, the therapeutic applications of the combination of testosterone plus DFMO or other Odc1 inhibitors as selective androgen/anabolic therapy that spares the prostate should be explored.

## Methods

### Expression and purification of human Fst288 (rFst)

Follistatin 288 (Fst288, amino acid residues 30–317) cDNA was cloned into Sal1/Xho1 site of pET30 and expressed with His-tag in *E. coli* BL21(DE3) after induction with IPTG. Cell lysate was centrifuged and the pellet was solubilized in a buffer containing 50 mm Tris–HCl, 8 m urea, 100 mm PMSF, pH 8.0. His-tagged Fst288 was purified with HisPur cobalt spin column (Thermo scientific, Rockford, IL, USA). Purified protein in elution buffer containing 8 m urea was diluted 1:4 with 200 mm Tris–HCl, pH 10, and 2 mm DTT and dialyzed against Tris buffer (10 mm Tris–HCl, 1 mm NaCl, pH 8.0) at 4°C. Purified protein was passed through Endotoxin Removal Gel and stored at −80°C in 20% glycerol.

Biological activity of Fst288 was verified by its ability to neutralize the growth inhibitory effect of activin on mouse MPC11 cells (Phillips *et al*., 1999). About 10^6^ viable cells were incubated in 0.1 mL DMEM supplemented with 10% fetal calf serum, penicillin and streptomycin, and 25 μm β-mercaptoethanol in a 96-well plate. Activin (Stemgent, MA, USA) was added and cells were allowed to grow for 48 h before the addition of 0.25 μCi of [^3^H] thymidine (6.7 Ci mmol^−1^) for another 24 h. Thymidine incorporation in cells was measured by Cerenkov counter.

### rFst administration

Eight-week-old C57BL6 mice (Jackson Laboratory, Bar Harbor, ME, USA) were housed in Laboratory Animal Science Center, using the protocols approved by the Institutional Animal Care and Use Committee of Boston University.

Animals were allowed to recover for 1 week before random assignment to one of four groups (*n* = 6 in each group) to receive 0, 10, 30, or 100 μg rFst in phosphate-buffered saline containing 20% glycerol by daily subcutaneous injection for 13 weeks. Body weights and lean and fat mass were measured weekly using EchoMRI-700 (Echo Medical System, Houston, TX, USA). Wet weights of muscle groups and dorsal prostate were measured after euthanasia.

### Microarray transcriptome analysis

Ten-week-old male mice were randomized to one of four groups: sham (control) operated, vehicle treated (*n* = 4), castrated, vehicle treated (*n* = 4), castrated+ testosterone treated (15 μg daily; *n* = 4), and castrated + rFst treated (15 μg daily; *n* = 4). Mice underwent castration under ketamine–xylazine, allowed to recover for 2 weeks, and injected subcutaneously with vehicle, 15 μg testosterone, or 15 μg rFst. The mice were euthanized after 24 h, and prostate and *levator ani* muscle were harvested.

Total RNA was prepared, and biotin-labeled cDNA was hybridized to Affymetrix Mouse 430 2.0 chips. Raw data were background-corrected, quantile-normalized, and summarized into log-transformed expression values using median polish according to RMA methodology (Irizarry *et al*., 2003).

Differential expression calculations were made using linear models to calculate a moderated *t*-statistic as detailed in Smyth (2004). A Benjamini–Hochberg correction was used to account for multiple hypothesis testing. All statistical calculations were made using limma package in R statistical program (http://bioconductor.org/packages/release/bioc/html/limma.html; http://www.rproject.org/;ftp://ftp.informatics.jax.org/pub/reports/index.html#go). A gene was classified as ‘androgen sensitive’ if it showed differential expression at a *q*-value < 0.2 under these two comparisons: (i) castrated versus sham-treated mice and (ii) testosterone-treated versus castrated mice. A gene was classified as ‘follistatin sensitive’ if it showed differential expression at a *q*-value < 0.2 under these two comparisons: (i) castrated versus sham-treated mice and (ii) rFst-treated versus castrated mice.

Gene Ontology term enrichment was used to identify biological processes over-represented in sets of genes identified through gene expression analysis. Gene annotations were limited to ‘biological process’ branch of the ontology and downloaded from the Mouse Genome Informatics (MGI) database. GO is a hierarchy, gaining biological specificity the further a term is away from the root. This parent–child relationship implies that a gene annotated to a child term is also annotated to the parent term, which in turn is annotated to all descendent terms. Accordingly, gene annotations were mapped to the GO and a gene was subsumed by all parent terms. This results in the root term, biological process, being annotated to all genes. The revised gene to GO term mapping was then filtered to remove GO terms with very high frequency and very low frequency. We required a term to have <300 and >5 associated genes for further analysis. For a set of genes, GO term enrichment for term *i* was calculated using hypergeometric function,

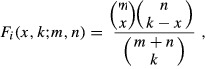
where *m* is the number of genes that term *i* is annotated to cover the entire dataset, *n* is the number of genes that are annotated with any GO term over the entire dataset, *k* is the number of genes that are selected, and *x* is the number of times term *i* is selected from *k* genes.

### Time-course experiments of testosterone administration in castrate mice

Eight-week-old C57BL6 mice (Jackson Laboratory) were housed in Laboratory Animal Science Center, using the protocols approved by the Institutional Animal Care and Use Committee of Brigham and Women’s Hospital. Animals were allowed to recover for 1 week before random assignment to one of three groups (*n* = 6 in each group): intact, castrated, or castrated + T to receive oil, oil, and oil + 30 μg T subcutaneously, respectively. The animals were euthanized at days 1, 3, 7, and 14 after the start of treatment. Wet weights of dorsal prostate were measured after euthanasia, and Odc1 expression was followed by qPCR analysis.

### *In vitro* studies

LNCaP cells, obtained from the American Type Culture Collection (Rockville, MD, USA), were maintained in 100-mm dishes (Corning, NY, USA) in RPMI 1640 supplemented with 5% fetal bovine serum and 1% antimycotic–antibiotic mix (Life Technologies, Rockville, MD, USA) at 37°C in 5% CO_2_. Cell cycle analysis was performed using FITC BrdU kit (BD Pharmingen, San Diego, CA, USA) according to the established protocols. Cells we pulsed with BrdU for 30 min, washed in phosphate-buffered saline containing 5% FBS, fixed and permeabilized with cytofix, and washed with Perm/Wash buffer. Subsequently, cells were treated with 30 mg of DNase for 1 h and stained with FITC-conjugated anti-BrdU antibody and 7-aminoactinomycin D (7-AAD) before flow cytometric analysis. DNA content was analyzed using a FACSscan flow cytometer. Primary prostate epithelial cells, obtained from Clonetics, were maintained in growth medium and synchronized for 24 h before the treatment with testosterone or rFst. PCNA mRNA expression was measured using quantitative PCR and normalized against the density of 18S ribosomes.

For the time-course experiments, LnCap cells were allowed to seed on T-25 flasks at 80% confluency overnight. Next day, cells were treated with vehicle (Con), 100 nm testosterone (Test), or 125 ng mL^−1^ rFst for different time points (0–24 h). Western blotting was performed according to the standard protocols. Typically, 40 μg total cell lysates was run on 4–15% SDS-PAGE (BioRad, Herculus, CA, USA), electrotransferred to PVDF membranes, and probed with Odc1 (1:500 dilutions, Hybridoma Bank) or α-tubulin (1:5000 dilutions, Chemicon) primary antibodies overnight. Membranes were incubated with HRP-conjugated mouse secondary antibody (1:1000) for 1 h at room temperature and developed using ECL reagent (Amersham, CA, USA). Densitometric scanning was performed to deduce relative Odc1 protein expression after normalization with α-tubulin.

### Testosterone and DFMO administration

Stock solutions of testosterone propionate (T) in sesame oil and DFMO in saline solution were stored at 4°C. The mice were divided into following groups: intact, castrate (CX), castrate co-administered with T and DFMO (Cx + T + DFMO), and castrate administered with testosterone (Cx + T). Each mouse received a subcutaneous injection of either 15 μg TP in 100 μL sesame oil or 100 μL sesame oil alone. This was followed by an intraperitoneal injection of 7.5 mg DFMO in 100 μL saline solution for Cx + T + DFMO group, or an intraperitoneal 100 μL saline injection for all other groups. Each afternoon, the DFMO or saline injection was repeated for all mice. Animals were euthanized on the 14th day and tissues were weighed.

## Supporting Information

**Fig. S1** Comparison of pathway expression for the prostate on a subnetwork centered on the Gene Ontology (GO) term, ‘polyamine biosynthetic process.’ **Fig. S2** Differential regulation of Odc1 mRNA expression in prostate tissues obtained from castrated mice after testosterone and Fst treatments. **Fig. S3** Recombinant Fst does not affect Odc1 protein expression in LnCaP cells. **Fig. S4** Odc1 expression is consistently upregulated by testosterone supplementation in castrated mice *in vivo*. **Fig. S5** Effect of castration and testosterone supplementation on prostate weights in mice.

**Table S1** Gene expression analysis for identifying androgen-sensitive and follistatin-sensitive genes in the prostate and *levator ani*, along with Gene Ontology (GO) term enrichment analysis for the associated sets of genes.
