# Supplementary material for: Combined administration of testosterone plus an ornithine decarboxylase inhibitor as a selective prostate-sparing anabolic therapy
Source: Aging Cell. 2013 Dec 4;13(2):303–10. doi: 10.1111/acel.12174 (PMC4331775; doi:10.1111/acel.12174)
Supplement: Supplementary file 1 — Fig. S1 Comparison of pathway expression for the prostate on a subnetwork centered on the Gene Ontology (GO) term, ‘polyamine biosynthetic process.’ Fig. S2 Differential regulation of Odc1 mRNA expression in prostate tissues obtained from castrated mice after testosterone and Fst treatments. Fig. S3 Recombinant Fst does not affect Odc1 protein expression in LnCaP cells. Fig. S4 Odc1 expression is consistently upregulated by testosterone supplementation in castrated mice in vivo. Fig. S5 Effect of castration and testosterone supplementation on prostate weights in mice. [file acel0013-0303-sd1.pdf]

Supplemental Figure SF1. Comparison of pathway expression for the prostate on a subnetwork centered on the Gene Ontology (GO) term, “polyamine biosynthetic process.”

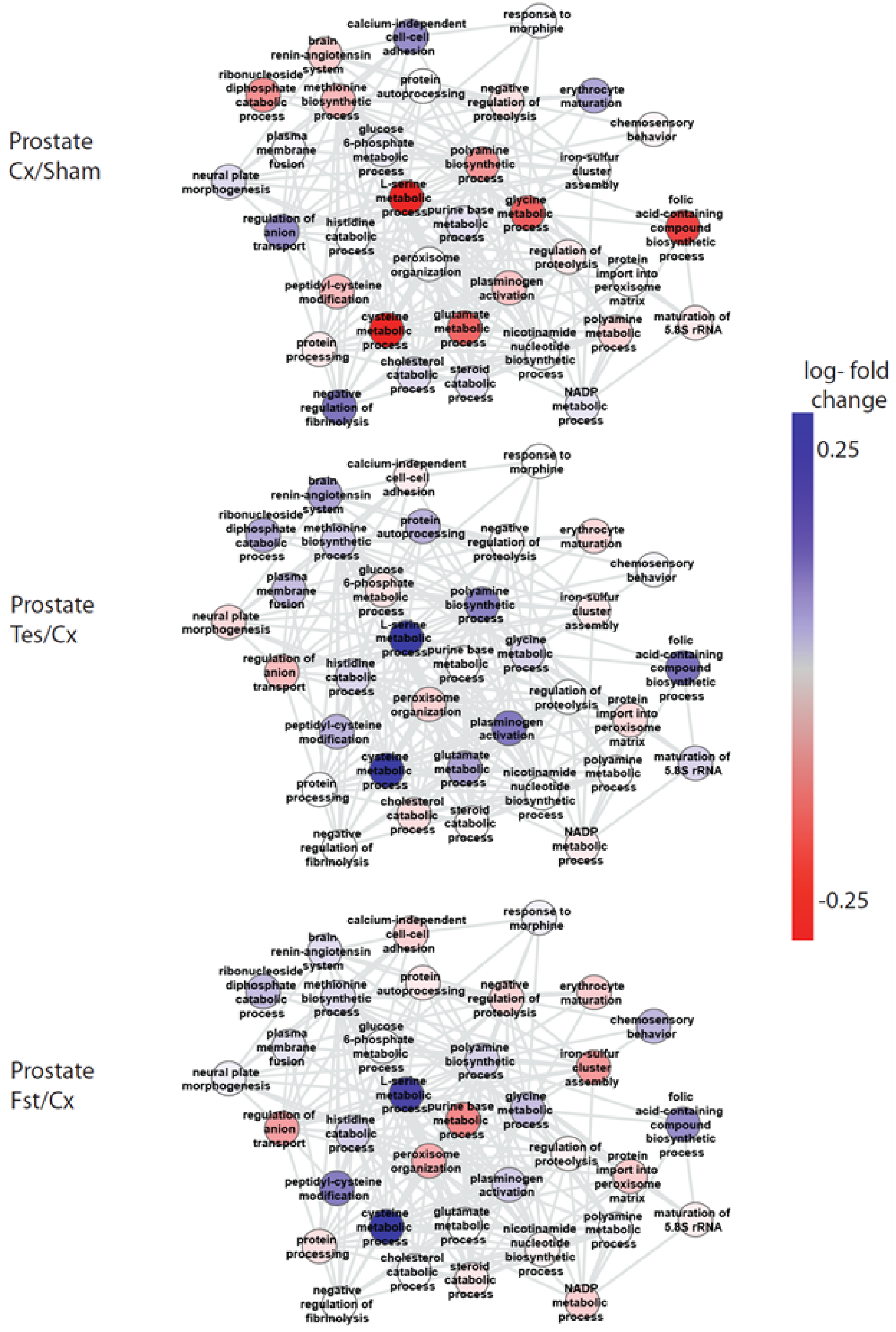

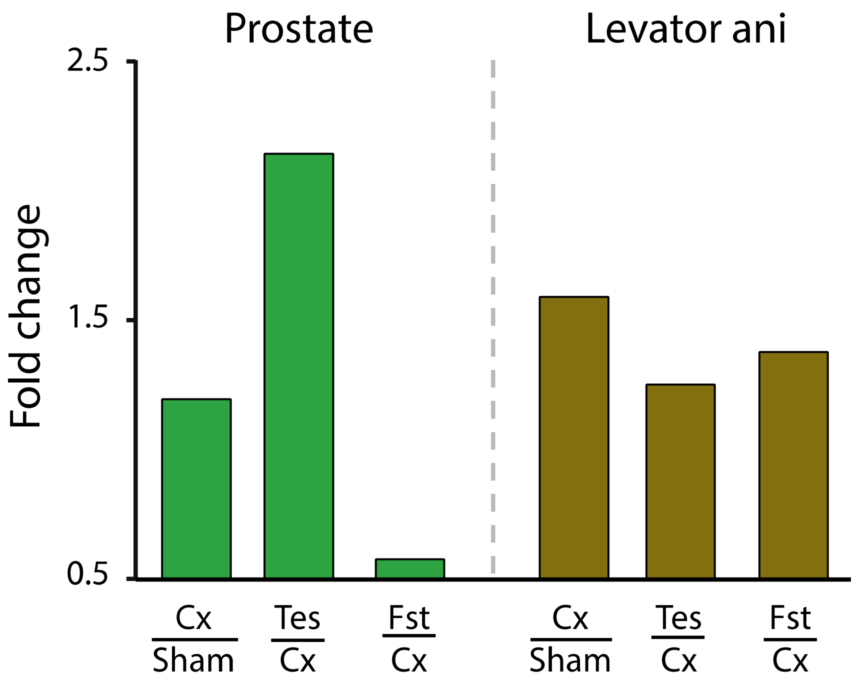

Figure S2. Differential regulation of Odc1 mRNA expression in prostate tissues obtained from castrated mice after testosterone and Fst treatments. qPCR analysis of the mRNA confirms that testosterone supplementation upregulates Odc1 while rFst treatment does not effect the expression in prostate.

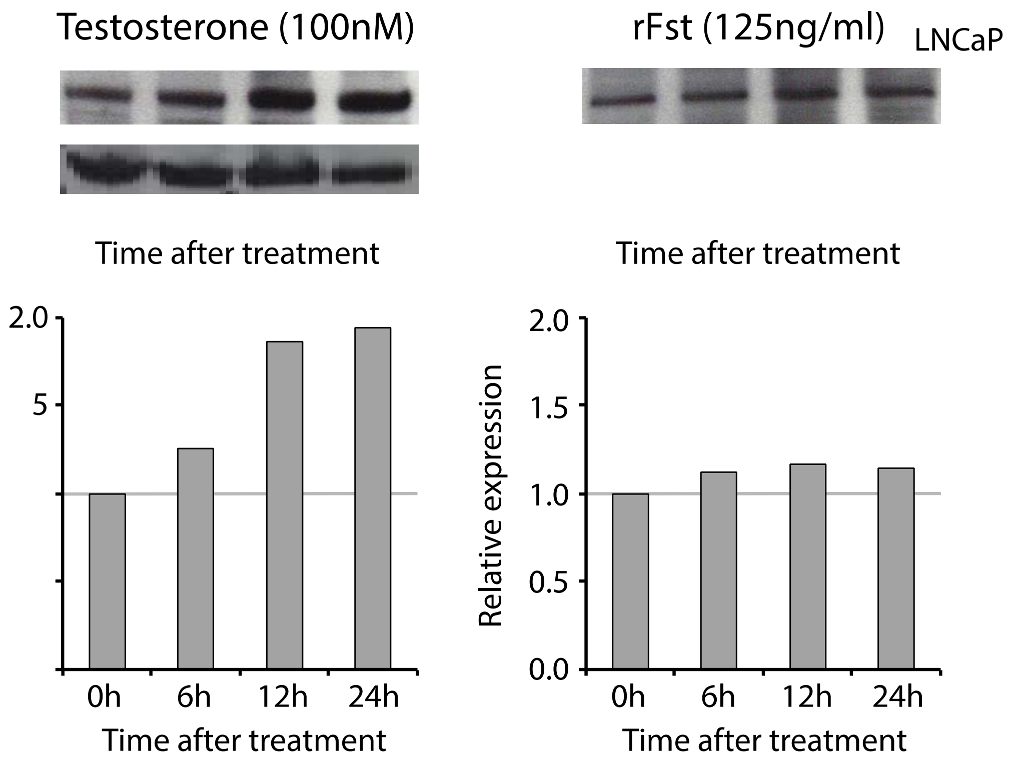

Figure S3: Recombinant Fst does not affect Odc1 protein expression in LnCaP cells. LnCap cell were treated with either testosterone (100 nM) or rFst (125 ng/mL0 and the lysates were probed for Odc1 expression at the depicted time points. The immublots confirm the selective induction of Odc1 expression by testosterone *in vitro*.

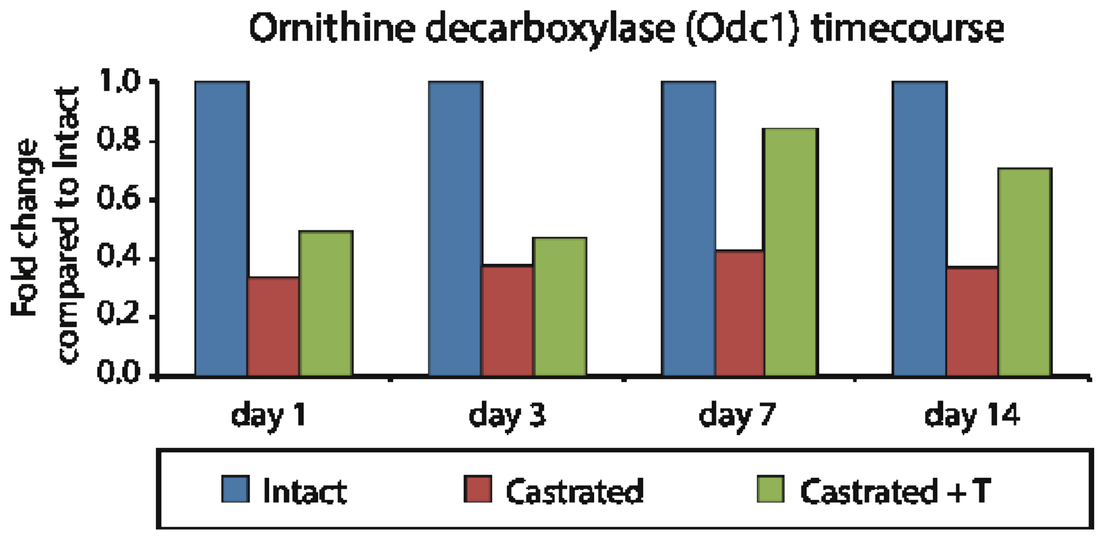

Figure S4: Odc1 expression is consistently upregulated by testosterone supplementation in castrated mice in-vivo. qPCR analysis of relative Odc1 mRNA expression in prostate tissues obtained from different treatment groups shows a time dependent increase in expression.

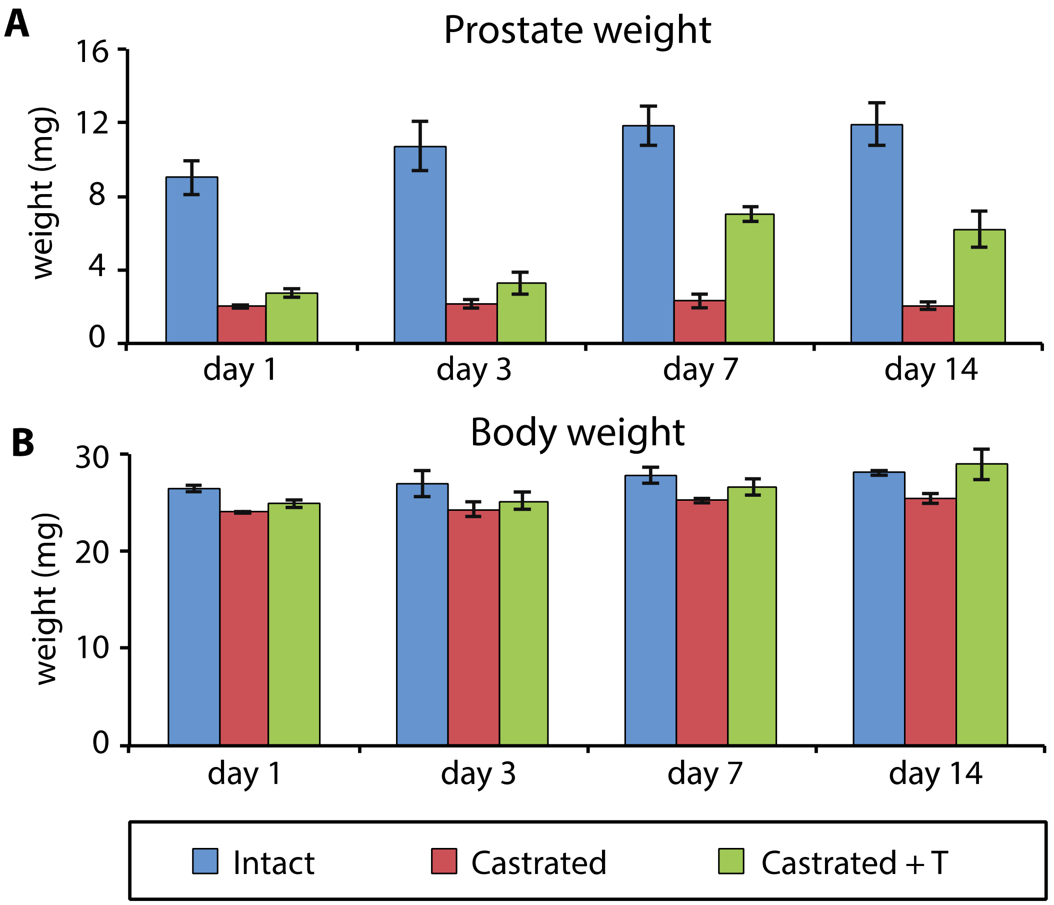

Figure S5. Effect of castration and testosterone supplementation on prostate weights in mice. Panel A shows the time-course of recovery of prostate weights in castrate mice upon testosterone administration. Panel B shows the corresponding body weights of animals in treatment groups.
