# Supplementary material for: Combined administration of testosterone plus an ornithine decarboxylase inhibitor as a selective prostate-sparing anabolic therapy
Source: Aging Cell. 2013 Dec 4;13(2):303–10. doi: 10.1111/acel.12174 (PMC4331775; doi:10.1111/acel.12174)
Supplement: Supplementary file 3 [file acel0013-0303-sd3.docx]

**Supplemental File Legends**

**Supplemental Table 1.** Gene expression analysis for identifying androgen-sensitive and follistatin-sensitive genes in the prostate and levator ani, along with GO term enrichment analysis for the associated sets of genes.

**Supplemental Table 1.1.** Gene expression analysis for the prostate. Log_2_ fold change and the Benjamini-Hochberg adjusted p-values are listed for all genes comparing the following groups: Castrated vs. Sham (untreated) mice, Testosterone-treated vs. Castrated mice, and follistatin-treated vs. Castrated mice. Genes are classified as androgen-sensitive and follistatin-sensitive.

**Supplemental Table 1.2** Gene Ontology (GO) term enrichment analysis for genes that are androgen-sensitive and not follistatin-sensitive in the prostate. Statistical significance is calculated according to the hypergeometric distribution.

**Supplemental Table 1.3** Gene expression analysis for the levator ani. Log_2_ fold change and the Benjamini-Hochberg adjusted p-values are listed for all genes comparing the following groups: Castrated vs. Shame (untreated) mice, Testosterone-treated vs. Castrated mice, and follistatin-treated vs. Castrated mice. Genes are classified as androgen-sensitive and follistatin-sensitive.

**Supplemental Table 1.4** Gene Ontology (GO) term enrichment analysis for genes that are androgen-sensitive and not follistatin-sensitive in the *levator ani*. Statistical significance is calculated according to the hypergeometric distribution.

**Supplemental Table 1.5** Gene Ontology (GO) term enrichment analysis for genes that are androgen- and follistatin-sensitive in the *levator ani*. Statistical significance is calculated according to the hypergeometric distribution.

**Supplemental Table 1.6** Gene Ontology(GO) term enrichment analysis for androgen- and follistatin-sensitive genes in the *levator ani*. Statistical significance is calculated according to the hypergeometric distribution
